# Which is the best treatment of pediatric upper urinary tract stones among extracorporeal shockwave lithotripsy, percutaneous nephrolithotomy and retrograde intrarenal surgery: a systematic review

**DOI:** 10.1186/s12894-019-0520-2

**Published:** 2019-10-23

**Authors:** Qing He, Kaiwen Xiao, Yuntian Chen, Banghua Liao, Hong Li, Kunjie Wang

**Affiliations:** Department of Urology, Institute of Urology (Laboratory of Reconstructive Urology), West China Hospital, Sichuan University, No. 37 Guo Xue Xiang, Chengdu, Sichuan 610041 People’s Republic of China

**Keywords:** Extracorporeal shockwave lithotripsy, Pediatric, Percutaneous nephrolithotomy, Retrograde intrarenal surgery, upper urinary tract stone

## Abstract

**Background:**

Although the indications of minimally invasive treatments for pediatric urolithiasis are similar to those in adults, it is still crucial to make the right treatment decision due to the special considerations of children. This review aims to evaluate the efficacy and safety of extracorporeal shockwave lithotripsy (SWL), percutaneous nephrolithotomy (PCNL), and retrograde intrarenal surgery (RIRS) in the management of pediatric upper urinary tract stones.

**Methods:**

EMBASE, PubMed, and the Cochrane Library were searched from their first available date to March 2018. The studies that meet the inclusive criteria were included. The efficacy and safety of the treatments were assessed by means of meta-analysis of the stone free rate (SFR), complication rate, effectiveness quotient (EQ) and secondary outcome indicators.

**Results:**

A total of 13 comparative studies were identified for data analysis. PCNL presented a significantly higher SFR compared with SWL. Similarly, the single-session SFR of RIRS was significantly higher than SWL. However, no significant difference was found between RIRS and SWL in the overall SFR. There was no significant difference between PCNL and RIRS in the SFR. Furthermore, no significant differences in complication rates were found among the three therapies. Compared with the other two treatments, PCNL had a longer operative time, fluoroscopy time and hospital stay. SWL had a shorter hospital stay, higher retreatment rate and auxiliary rate in comparison with the other two treatments. The present data also showed that PCNL presented a higher EQ than the other two treatments, and RIRS had a lower efficiency than SWL and PCNL. In the subgroup analysis of pediatric patients with stone ≤20 mm, the comparative results were similar to those described above, except for the higher complication rate of PCNL than SWL.

**Conclusions:**

Although SWL as an outpatient procedure provides shorter hospital stay and reduces operative time, it has a lower SFR and higher retreatment rate than the other two treatments. PCNL exhibits a higher SFR and EQ than SWL; nevertheless, it has a longer operative time and fluoroscopy time than the other two procedures. RIRS offers a similar SFR as PCNL but a lower efficiency than PCNL.

## Background

Pediatric urolithiasis is an important global medical issue, particularly in regards to the selection of different treatments by care providers. The primary risk factors of forming stones among children include geographical conditions, climates, and diet customs. Additionally, the morbidity is opposite to the level of economic development; the morbidity is 1 to 5% in developed nations and 5 to 15% in developing nations [1]. Nevertheless, rates of pediatric urolithiasis have increased in developed countries. Extracorporeal shockwave lithotripsy (SWL) has long been considered as the first-line therapy for pediatric urolithiasis less than 20 mm [2]. There is a growing trend in the management of pediatric urolithiasis with endourologic procedures due to the technological advances and miniaturization of instruments. SWL, percutaneous nephrolithotomy (PCNL), and retrograde intrarenal surgery (RIRS) have become standard therapeutic options for adult urinary stones and can be extended to pediatric stones.

SWL is still the first choice for pediatric renal stones since it is the least-invasive approach for managing pediatric urolithiasis. The indications of SWL for pediatric urolithiasis are similar to those of adults, according to the European Association of Urology (EAU) guideline, and the stone fragments are more easily passed by pediatric patients than adult patients [3]. Similarly, the American Urological Association (AUA) guideline recommends that clinicians offer SWL or RIRS as first-line therapy for pediatric patient with a total renal stone burden under 20 mm [4]. However, the high retreatment rates and the potential biological effects on the immature kidneys and adjacent tissues may limit the range of application of SWL.

Age is not a limiting factor, since there are appropriately sized PCNL instruments for children, and this approach has even been reported in a 5-month-old infant [5, 6]. In children, PCNL is recommended to manage renal stones larger than 20 mm [3, 4], especially multiple renal stones. The most common complications are fever and bleeding with some serious cases requiring blood transfusions to prevent ischaemia. The necessary radiation exposure and its effects on pediatric renal function remain controversial [7].RIRS is considered as an ideal choice for medium and large-sized pediatric renal or ureteric stones. With their smaller diameter, excellent optical properties, relatively wider range of motion, and the multiple secure and effective lithotripsy techniques available, increasing numbers of urologists prefer to treat pediatric upper urinary tract stones using ureteroscopy [8, 9]. Nevertheless, ureteroscopy, especially flexible ureteroscopy, is quite a challenge for clinicians who lack the necessary training. In addition, the high purchase and maintenance costs of RIRS result in this technique not being available in every medical centre.

In adults, any of the above three procedures could be recommended to patients with stones smaller than 20 mm located in the renal pelvis and upper or middle calices [3]. Although the indications for treatment in pediatric urolithiasis are similar to those of adults, the unique considerations, such as a smaller anatomical structure and immature kidneys, make it vital to identify the most effective and safest procedure for children. To evaluate the efficacy and safety of SWL, PCNL, and RIRS in the management of pediatric upper urinary tract stones, we performed a systematic review, synthesizing the available high-level studies.

## Methods

### Study identification

Literature databases including EMBASE, PubMed, and the Cochrane Library, were searched from their first available date to March 2018. The first search procedure was performed to identify all relevant trials retrieved using the following search terms: (extracorporeal shock wave lithotripsy/ESWL/SWL or percutaneous nephrolithotomy/percutaneous lithotripsy/PCNL or Retrograde intrarenal surgery/RIRS/flexible ureterorenoscopy/flexible ureteroscopy/URS/FURS) and (Pediatric/pediatrics/child/children). The language was restricted to English. Scanning the reference lists of the selected articles to identify additional articles was also conducted.

### Inclusion and exclusion criteria

The inclusion criteria were: 1) comparative studies evaluating the efficacy of SWL versus PCNL, SWL versus RIRS, PCNL versus RIRS, or SWL versus PCNL versus RIRS in the treatment of pediatric renal or upper ureteral calculi; and 2) the stone-free status was evaluated postoperatively by KUB film and/or ultrasonography. In addition, for the exclusion criteria were: 1) abstracts, comments, reviews, conference papers, or systematic reviews; and 2) inclusion of children with distal ureteral stones, uncontrolled coagulation disorders, active urinary tract infections, obstructive urinary tract anomalies, severe hydronephrosis or kidney failure (GFR < 15 mL/min). Duplicated studies were included in our research, however, only the latest data were assessed in our review.

### Data extraction and outcome measurement

Two investigators reviewed the titles and abstracts identified by the search strategies. The following data were extracted from each study if available by using a Microsoft Excel worksheet: first author’s name, year of publication, mean age of the patients, type of stones, stone sizes, stone free rate (SFR), complications, operative time, length of hospital stay, need for auxiliary procedures, and retreatments. We graded the complications according to the Clavien-Dindo classification of surgical complications [10]. To increase the comparability among these treatment modalities, the effectiveness quotients (EQs) were calculated according to the equation reported by Clayman and colleagues [11]. Dichotomous data were classified into two-by-two tables. For continuous data, available summary estimates per group (mean, changes in means) and measures of variability (standard deviation [SD], 95% confidence interval [CI]) were extracted.

### Evaluation of study quality

The levels of evidence (LE) of all included studies were assessed by the Oxford Centre for Evidence Based Medicine-Levels of Evidence [12]. The methodological quality of the studies was evaluated according to the Modified Jadad Scale for randomized controlled trials (RCTs) [13] and the Newcastle-Ottawa Scale (NOS) for nonrandomized controlled trials [14].

### Statistical analysis

The efficacy and safety of SWL, PCNL, and RIRS in the treatment of pediatric renal or ureteral calculi was accessed by the OR and mean difference with corresponding 95% CI under the paired comparisons among the three treatment techniques. The OR value was calculated by using the Z test. In addition, if *p* < 0.05, the difference was considered as statistically significant. When comparing the risk of continuous variables, the mean values and SDs are necessary for the pooled data. The random-effects model was used to generate the most conservative estimate. The chi-squared-based Q test was used to assess the heterogeneity with the significance level set to *p* < 0.10. Subgroup analysis was conducted according to stone size ≤20 mm to increase the comparability of these procedures and reduce the heterogeneity of results. All of the statistical analyses were performed using the RevMan5.3 software.

## Results

### Study identification and characteristics

**Fig. 1:**
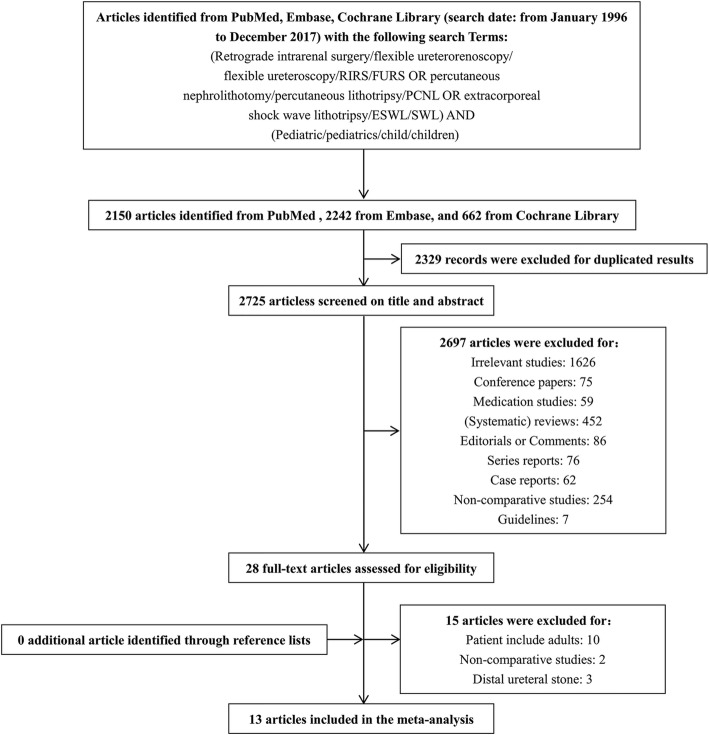
PRISMA flow diagram of study selection

The process of the selection of studies included in this review is summarized in Fig. 1. In total, 13 articles were included in this quantitative synthesis [15–27]. There were 3 randomized controlled trials [17, 21, 24] (RCTs) (LE: 2b), 1 prospective case controlled study [16] (LE: 3b) and 9 retrospective case controlled studies [15, 18–20, 22, 23, 25–27] (LE: 3b) (Table 1). Seven nonrandomized studies were relatively high (NOS: 6 of 9 points) and 3 nonrandomized studies were medium (NOS: 5 of 9 points) in methodological quality. The methodological quality of the 3 RCTs were relatively low (Modified Jadad Scale: 2 of 3 points and 1 of 2 points) due to a lack allocation concealment and a lack blinding methods.

**Table 1:** Summary of publications included in the meta-analysis

Study	Study period	Country	Study design	Cases, n			LE	Study quality
				SWL	PCNL	RIRS		
Zeng et al [15]	2005–2011	China	RCCS	22	24	–	3b	6^b^
Wadhwa et al [16]	2005–2005	India	PCCS	8	6	–	3b	5^b^
Kumar et al [17]	2012–2013	India	RCT	106	106	–	2b	3^a^
Hatipoglu et al [18]	2010–2012	Turkey	RCCS	108	37	–	3b	6^b^
Shokeir et al [19]	1995–2004	Egypt	RCCS	91	75	–	3b	6^b^
ElSheemy et al [20]	2010–2014	Egypt	RCCS	64	54	–	3b	6^b^
Mokhless et al [21]	3 months follow-up	Egypt	RCT	30	–	30	2b	3^a^
Freton et al [22]	2000–2014	France	RCCS	100	–	46	3b	5^b^
Resorlu et al [23]	2008–2011	Turkey	MRCCS	–	106	95	3b	6^b^
Saad et al [24]	2011–2014	Egypt	RCT	–	20	18	2b	2^a^
Bas et al [25]	2011–2015	Turkey	RCCS	–	45	36	3b	6^b^
Sen et al [26]	2015–2016	Turkey	RCCS	–	25	23	3b	5^b^
Pelit et al [27]	2013–2016	Turkey	RCCS	–	45	32	3b	6^b^

a.Modified Jadad scale (score from 0 to 7); b. Newcastle-Ottawa scale (score from 0 to 9). *SWL* Extracorporeal shockwave lithotripsy, *PCNL* Percutaneous nephrolithotomy, *RIRS* Retrograde intrarenal surgery, *LE* Level of evidence, *RCCS* Retrospective case control study, *PCCS* Prospective case control study, *RCT* Randomized control trail, *MRCCS* Multicenter retrospective case control study

A total of 529 SWL cases (50.38%), 241 PCNL cases (22.95%), and 280 RIRS cases (26.67%) were included. The baseline characteristics of the children are summarized in Table 2.

**Table 2:** Summary of pediatric patients’ baseline of included studies

Author	Treatment	Age, years	Sex		Stone location, n (units)					Side		Stone size, mm
					Pelvis	Caliceal	Pelvis + caliceal	Staghorn stone				
		(Mean ± SD)	Male	Female				Partial	Complete	Unilateral	Bilateral	(Mean ± SD)
Zeng et al [15]	SWL	1.96 ± 0.55	17	5	5(5)	0(0)	2(2)	15(15)	0(0)	22	0	21.7 ± 1.7
	PCNL	1.92 ± 0.8	15	9	6(7)	0(0)	3(4)	12(12)	3(3)	24	1	21.4 ± 3.5
Wadhwa et al [16]	SWL	3~12	11	3	8(9)	0(0)	0(0)	0(0)	0(0)	NA		220.4^a^
	PCNL	5.9			NA(4)	0(0)	NA(4)	1(1)	0(0)			1393.4^a^
Kumar et al [17]	SWL	10.7 ± 1.3	52	54	NA					106	0	12.9 ± 1.3
	PCNL	10.3 ± 1.2	51	55						106	0	12.7 ± 1.2
Hatipoglu et al [18]	SWL	5.91 ± 4.03	61	47	50	58	0(0)	0(0)	0(0)	108	0	11.32 ± 2.84
	PCNL	8.43 ± 4.84	15	22	10	27	0(0)	0(0)	0(0)	37	0	14.78 ± 5.39
Shokeir et al [19]	SWL	6.4 ± 1.4	50	41	NA(70)	NA(11)	NA(12)	0(0)	0(0)	89	2	13.9 ± 4.2
	PCNL	6.6 ± 1.2	45	30	NA(52)	NA(14)	NA(16)	0(0)	0(0)	68	7	14.4 ± 3.1
ElSheemy et al [20]	SWL	4.06 ± 0.96	44	20	52	12	0(0)	0(0)	0(0)	64	0	14.87 ± 4.05
	PCNL	3.84 ± 1.44	33	21	39	15	0(0)	0(0)	0(0)	54	0	15.98 ± 4.31
Mokhless et al [21]	SWL	1~6	40	20	15(NA)	5(NA)	10(NA)	0(0)	0(0)	60	0	10–20
	RIRS	2.4 ± 1.3			17(NA)	7(NA)	6(NA)	0(0)	0(0)			10–20
Freton et al [22]	SWL	6.7 ± 0.6	NA		Upper tract urinary stones (upper ureter or kidney)					NA		19.5 ± 1.5
	RIRS	9.1 ± 0.9										21.6 ± 2.0
Resorlu et al [23]	PCNL	9.6 ± 4.9	56	50	36(NA)	70(NA)	0(0)	0(0)	0(0)			23.7 ± 4.42
	RIRS	9.3 ± 5.2	53	42	29(NA)	66(NA)	0(0)	0(0)	0(0)			14.3 ± 3.81
Saad et al [24]	PCNL	6.93 ± 3.55	14	8	2(2)^b^		17(17)^c^	3(3)		22	0	>20
	RIRS	6.44 ± 4.84	14	7	5(5)^b^		11(11)^c^	5(5)		21	0	>20
Bas et al [25]	PCNL	5.62 ± 4.50	23	22	27	18	0(0)	0(0)	0(0)	45	0	13.97 ± 3.46
	RIRS	8.39 ± 4.72	15	21	14	22	0(0)	0(0)	0(0)	36	0	12.80 ± 3.03
Sen et al [26]	PCNL	4 ± 2.3	NA		1	24	0(0)	0(0)	0(0)	NA		12.2 ± 2.8
	RIRS	10.9 ± 3			0	23	0(0)	0(0)	0(0)			13.7 ± 3.5
Pelit et al [27]	PCNL	3.71 ± 1.89	24	21	Renal stones					NA		21.06 ± 5.61
	RIRS	3.65 ± 1.95	17	15								19.30 ± 4.21

a. mm2, b. single stone, c. multiple stones. *SWL* Extracorporeal shockwave lithotripsy, *PCNL* Percutaneous nephrolithotomy, *RIRS* Retrograde intrarenal surgery, *NA* Not available

### Meta-analysis outcomes

#### Overall SFR and single-session SFR

**Fig. 2:**
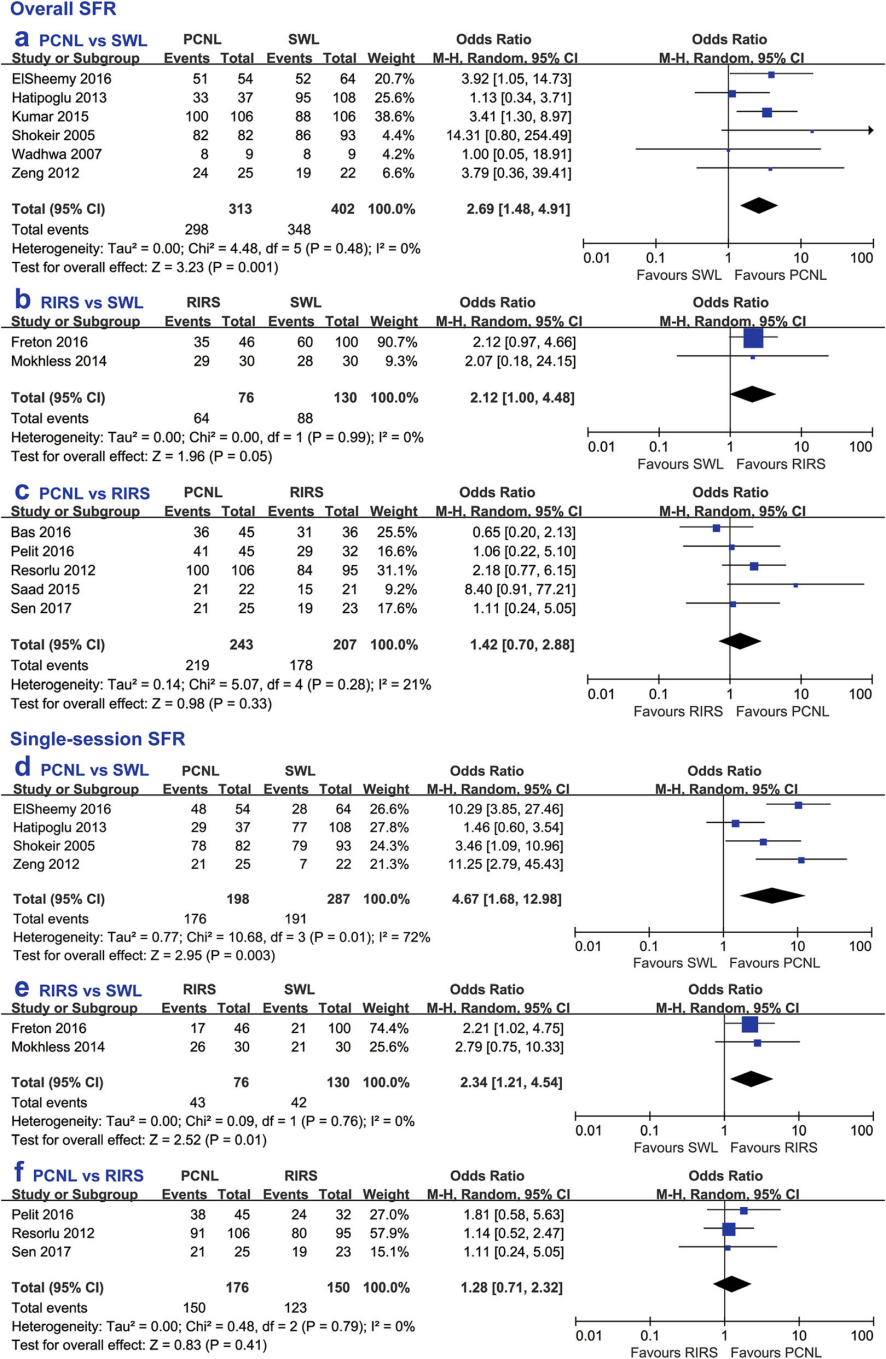
Forest plot comparing overall SFR between (**a**) PCNL and SWL, **b** RIRS and SWL, **c** PCNL and RIRS, and single-session SFR between (**d**) PCNL and SWL, **e** RIRS and SWL, **f** PCNL and RIRS

All the included studies defined stone free as no residual fragments except one study [22] that defined stone free as fragments no larger than 4 mm. PCNL presented a significantly higher overall SFR (OR 2.69, 95% CI 1.48 to 4.91, *p* = 0.001) and single-session SFR (OR 4.67, 95% CI 1.68 to 12.98, *p* = 0.003) than SWL. Similarly, the single-session SFR of RIRS was significantly higher than SWL (OR 2.34, 95% CI 1.21 to 4.54, *p* = 0.01), but there was no significant difference in the overall SFR between RIRS and SWL (OR 2.12, 95% CI 1.00 to 4.48, *p* = 0.05). Furthermore, PCNL had similar effects in the overall SFR and single-session SFR in comparison with RIRS (OR 1.42, 95% CI 0.70 to 2.88, *p* = 0.33; OR 1.28, 95% CI 0.71 to 2.32, *p* = 0.41). (Fig. 2).

#### Complication rate, minor (Clavien-Dindo I–II) complication rate and major (Clavien-Dindo III–IV) complication rate

**Fig. 3:**
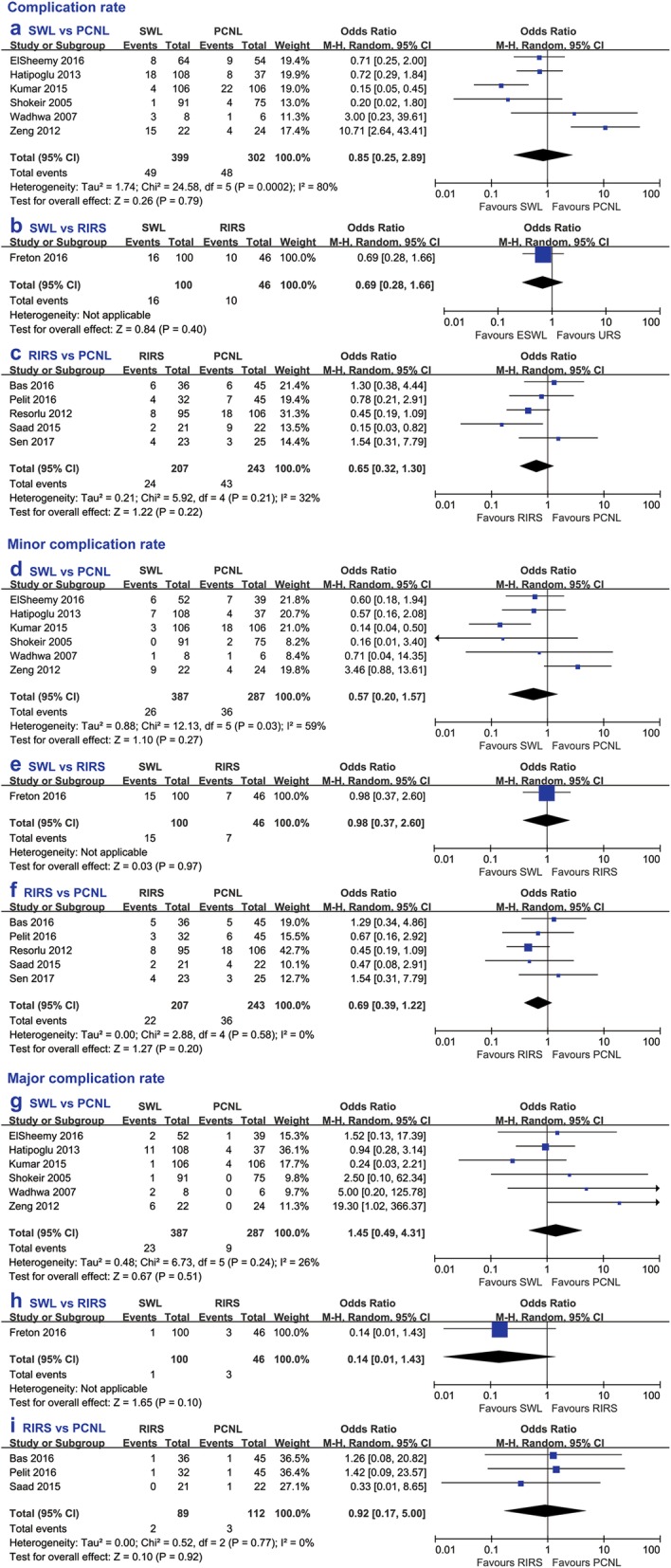
Forest plot comparing complication rate between (**a**) SWL and PCNL, **b** SWL and RIRS, **c** RIRS and PCNL, minor complication rate between (**d**) SWL and PCNL, **e** SWL and RIRS, **f** RIRS and PCNL, and major complication rate between (**g**) SWL and PCNL, **h** SWL and RIRS, **i** RIRS and PCNL

Intriguingly, no significant difference in the complication rate was found among the three procedures (SWL vs PCNL OR 0.85, 95% CI 0.25 to 2.89, *p* = 0.79; SWL vs RIRS OR 0.69, 95% CI 0.28 to 1.66, *p* = 0.40; and RIRS vs PCNL OR 0.65, 95% CI 0.32 to 1.30, *p* = 0.22). Furthermore, we analysed the postoperative complications according to grade. The results showed, similar to the overall complication rates, the minor (Clavien-Dindo I**–**II) complication rates (SWL vs PCNL OR 0.57, 95% CI 0.20 to 1.57, *p* = 0.27; SWL vs RIRS OR 0.98, 95% CI 0.37 to 2.60, *p* = 0.97; and RIRS vs PCNL OR 0.69, 95% CI 0.39 to 1.22, *p* = 0.20) and the major (Clavien-Dindo III**–**IV) complication rates (SWL vs PCNL OR 1.45, 95% CI 0.49 to 4.31, *p* = 0.28; SWL vs RIRS OR 0.14, 95% CI 0.01 to 1.43, *p* = 0.10; and RIRS vs PCNL OR 0.92, 95% CI 0.17 to 5.00, *p* = 0.92) were not significantly different among these three procedures (Fig. 3). The detailed complications reported by the included studies are summarized in Table 3.

**Table 3:** Summary of detailed complications of included studies

Author	Treatment	Complications, n(%)										
		Total	Renal colic	Fever	Urinary infection	Hematuria	Uroepsis	Steinstrasse	Blood transfusion	Ureteral injury	Perforation	Others
Zeng et al [15]	SWL	15(68.19)	5(22.73)	4(18.19)	–	1(4.55)	–	4(18.19)	–	–	–	1(4.55)
	PCNL	4(16)	–	4(16)	–	–	–	–	–	–	–	–
Wadhwa et al [16]	SWL	3(37.5)	–	1(12.5)	–	–	–	2(25)	–	–	–	–
	PCNL	1(16.7)	–	1(16.7)	–	–	–	–	–	–	–	–
Kumar et al [17]	SWL	4(3.7)	2(1.85)	–	1(0.93)	1(0.93)	–	–	–	–	–	–
	PCNL	22(20.75)	5(4.72)	–	9(8.49)	8(7.55)	–	–	–	–	–	–
Hatipoglu et al [18]	SWL	18(16.67)	7(6.6)	–	–	–	–	11(10.38)	–	–	–	–
	PCNL	8(21.62)	4(10.81)	–	–	–	–	1(2.70)	–	–	–	3(8.11)
Shokeir et al [19]	SWL	1(1.1)	–	–	–	–	–	1(1.1)	–	–	–	–
	PCNL	4(5.41)	–	2(2.7)	–	–	–	–	1(1.35)	–	1(1.35)	–
ElSheemy et al [20]	SWL	8(15.4)	–	4(7.7)	4(7.7)	–	–	4(7.7)	–	–	–	–
	PCNL	8(20.5)	–	7(17.94)	2(5.1)	–	–	–	–	–	1(2.6)	2(5.1)
Mokhless et al [21]	SWL	NA^a^	–	–	–	–	–	–	–	–	–	–
	RIRS	NA^a^	–	–	–	–	–	–	–	–	–	–
Freton et al [22]	SWL	16(16)	11(11)	1(1)	2(2)	–	–	–	–	–	–	2(2)
	RIRS	10(21.7)	–	–	3(6.5)	1(2.2)	–	1(2.2)	–	–	–	5(10.9)
Resorlu et al [23]	PCNL	18(17)^b^	–	–	–	–	–	–	–	–	–	–
	RIRS	8(8.4)^b^	–	–	–	–	–	–	–	–	–	–
Saad et al [24]	PCNL	9(40.9)	–	4(18.2)	–	–	–	–	3(13.6)	–	–	2(9.1)
	RIRS	2(9.5)	–	2(9.5)	–	–	–	–	–	–	–	–
Bas et al [25]	PCNL	6(13.3)	4(8.9)	1(2.2)	1(2.2)	–	–	–	–	–	–	–
	RIRS	6(16.7)	2(5.6)	2(5.6)	2(5.6)	–	–	–	–	–	–	–
Sen et al [26]	PCNL	3(12)	3(12)	2(8)	–	–	–	–	–	–	–	–
	RIRS	4(17.3)	4(17.3)	4(17.3)	–	–	1(4.3)	–	–	–	–	–
Pelit et al [27]	PCNL	7(15.5)	–	–	2(4.4)	–	–	–	3(6.7)	–	1(2.2)	1(2.2)
	RIRS	4(12.5)	–	–	3(9.4)	–	–	–	–	1(3.1)	–	–

a. No major complications; b. No detailed complications. *SWL* Extracorporeal shockwave lithotripsy, *PCNL* Percutaneous nephrolithotomy, *RIRS* Retrograde intrarenal surgery

#### Operative time, fluoroscopy time and hospital stay

**Fig. 4:**
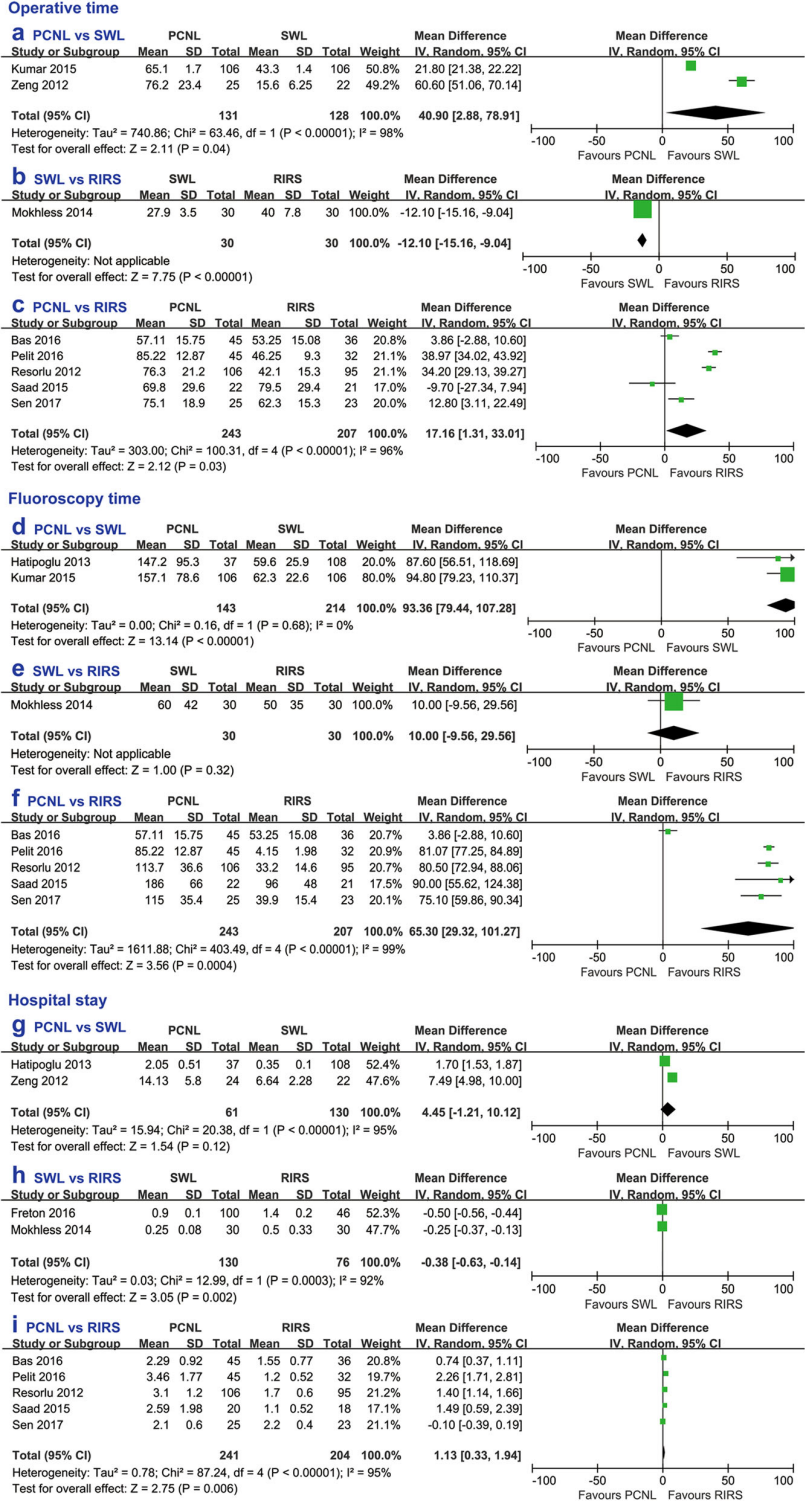
Forest plot comparing operative time between (**a**) PCNL and SWL, **b** SWL and RIRS, **c** PCNL and RIRS, fluoroscopy time between (**d**) PCNL and SWL, **e** SWL and RIRS, **f** PCNL and RIRS, and hospital stay between (**g**) PCNL and SWL, **h** SWL and RIRS, **i** PCNL and RIRS

Compared with the other two procedures, PCNL had a longer operative time, fluoroscopy time and hospital stay than SWL and RIRS (Fig. 4). In addition, SWL had a significantly shorter operative time (weighted mean difference [WMD] − 12.10, 95% CI − 15.16 to − 9.04, *p* < 0.00001) and hospital stay (WMD − 0.38, 95% CI − 0.63 to − 0.14, *p* = 0.002) than RIRS. There was no significant difference in fluoroscopy time between SWL and RIRS (WMD 10.00, 95% CI − 9.56 to 29.56, *p* = 0.32). (Fig. 4).

#### Retreatment rate and auxiliary procedure rate

**Fig. 5:**
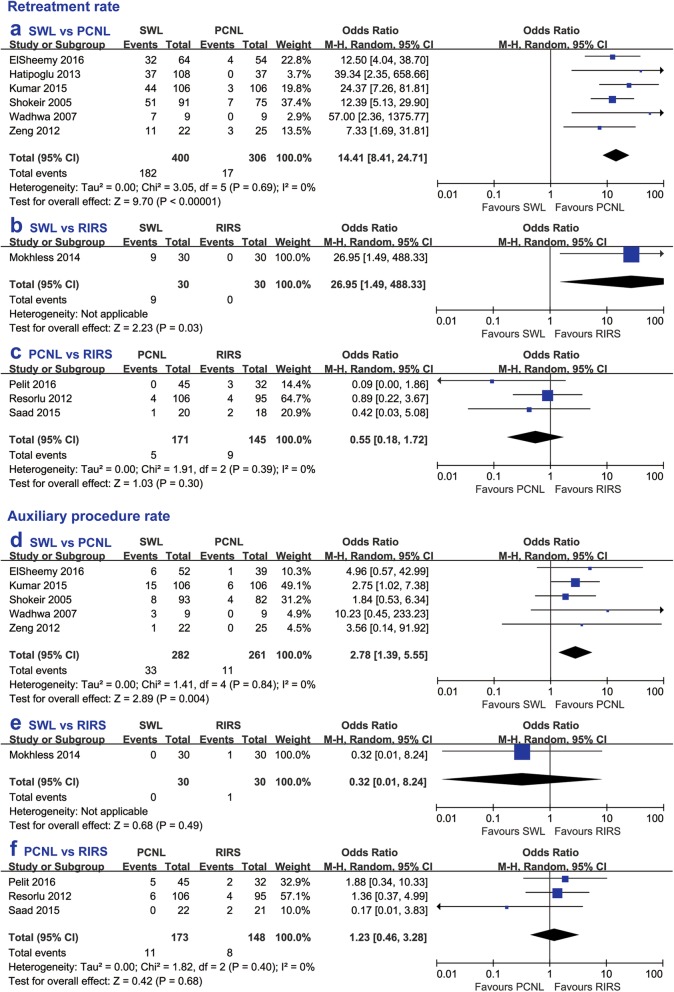
Forest plot comparing retreatment rate between (**a**) SWL and PCNL, **b** SWL and RIRS, **c** RIRS and PCNL, and auxiliary procedure rate between (d) SWL and PCNL, **e** SWL and RIRS, **f** RIRS and PCNL

SWL exhibited a significantly higher retreatment rate than PCNL (OR 14.41, 95% CI 8.41 to 24.71, *p* < 0.00001) and RIRS (OR 26.95, 95% CI 1.49 to 488.33, *p* = 0.03), while the retreatment rate between PCNL and RIRS (OR 0.55, 95% CI 0.18 to 1.71, *p* = 0.30) did not show any significant difference. The auxiliary procedure rate of RIRS had no significant difference compared with the other two treatments. Furthermore, SWL had a significantly higher auxiliary procedure rate compared with PCNL (OR 2.78, 95% CI 1.39 to 5.55, *p* = 0.004) (Fig. 5).

#### EQ

**Fig. 6:**
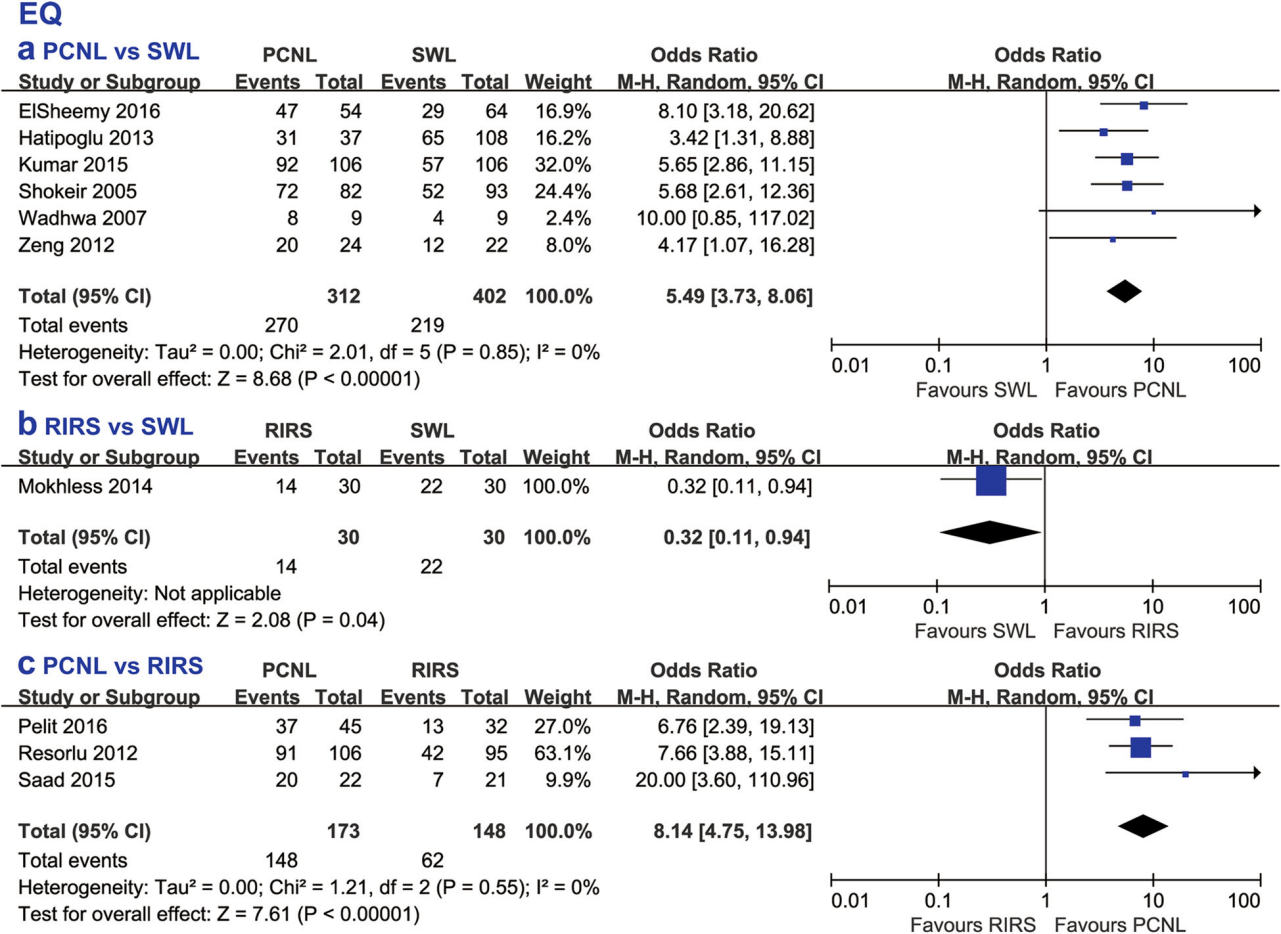
Forest plot comparing effectiveness quotient (EQ) between (**a**) PCNL and SWL, **b** RIRS and SWL, **c** PCNL and RIRS

According to the calculated EQs (Fig. 6), PCNL had a higher efficiency than SWL (OR 5.49, 95% CI 3.73 to 8.06, *p* < 0.00001) and RIRS (OR 8.14, 95% CI 4.75 to 13.98, *p* < 0.00001). Only one study [20], which compared RIRS to SWL, provided enough data to identify a lower EQ of RIRS than SWL (OR 0.32, 95% CI 0.11 to 0.94, *p* = 0.04).

### Subgroup analyses

#### PCNL and SWL

**Fig. 7:**
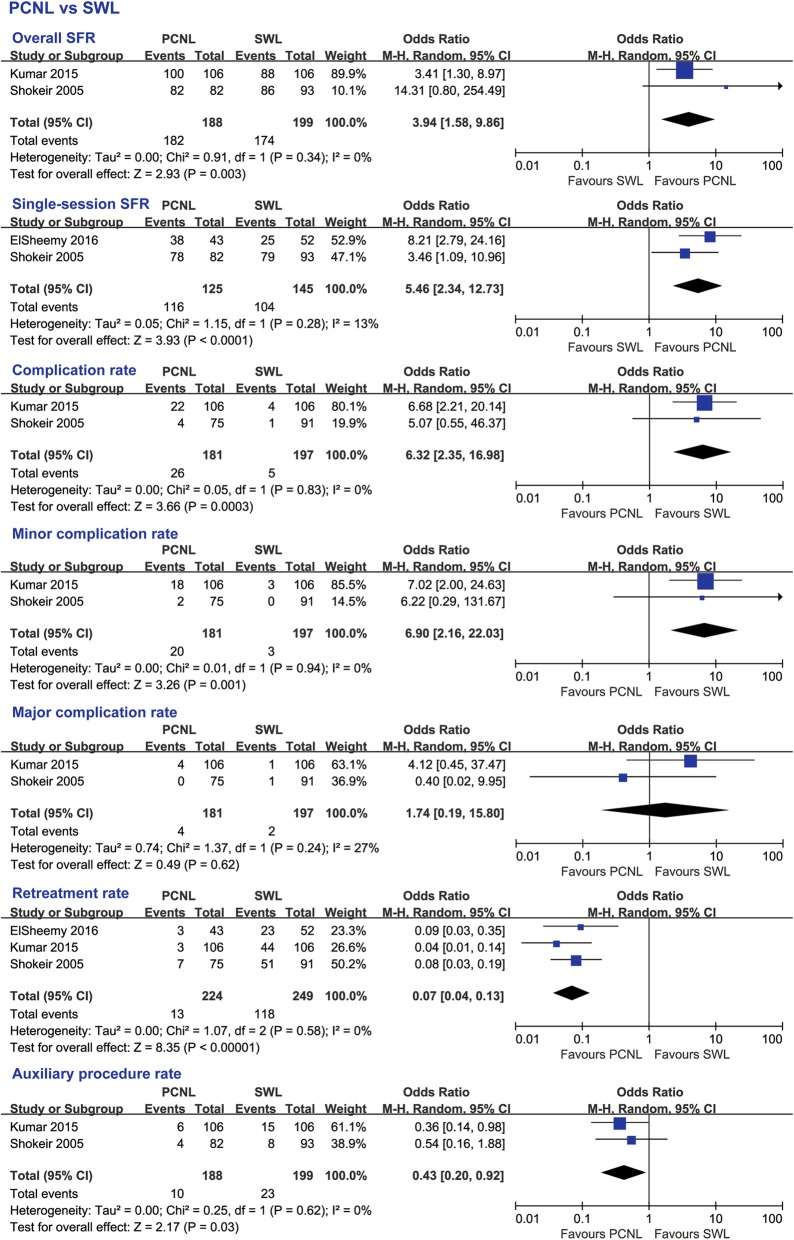
Subgroup analyses results of PCNL vs SWL

Obviously, PCNL still presented a significantly higher overall SFR (OR 3.94, 95% CI 1.58 to 9.86, *p* = 0.003) and single-session SFR (OR 5.46, 95% CI 2.34 to 12.73, *p* < 0.0001) than SWL in the treatment of pediatric patients with upper urinary tract stone of ≤20 mm (Fig. 7). Except for the approximate major complication rate (OR 1.74, 95% CI 0.19 to 15.80, *p* = 0.62), PCNL had both a significantly higher complication rate (OR 6.32, 95% CI 2.35 to 16.98, *p* = 0.0003) and a higher minor complication rate (OR 6.90, 95% CI 2.16 to 22.03, *p* = 0.001) (Fig. 7). However, pediatric patients with a stone size ≤20 mm who were treated with SWL were more likely to have multiple operations (PCNL vs SWL: OR 0.07, 95% CI 0.04 to 0.13, *p* < 0.00001) and additional procedures (PCNL vs SWL: OR 0.43, 95% CI 0.20 to 0.92, *p* = 0.03) (Fig. 7).

#### RIRS and SWL

**Fig. 8:**
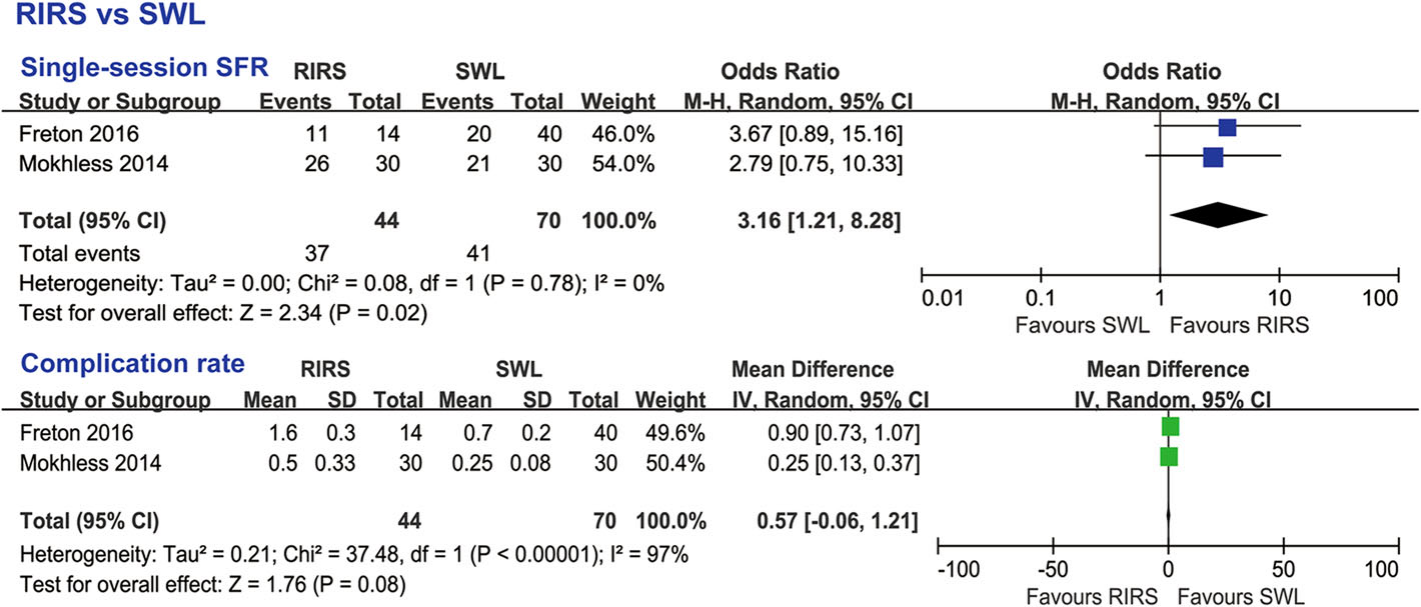
Subgroup analyses results of RIRS vs SWL

RIRS had a significantly higher single-session SFR than SWL (OR 3.16, 95% CI 1.21 to 8.28, *p* = 0.02) (Fig. 8). Although SWL was always conducted as an outpatient procedure, the results of the subgroup analyses did not show statistically significant differences of hospital stay between RIRS and SWL (WMD 0.57, 95% CI − 0.06 to 1.21, *p* = 0.08) (Fig. 8).

#### PCNL and RIRS

**Fig. 9:**
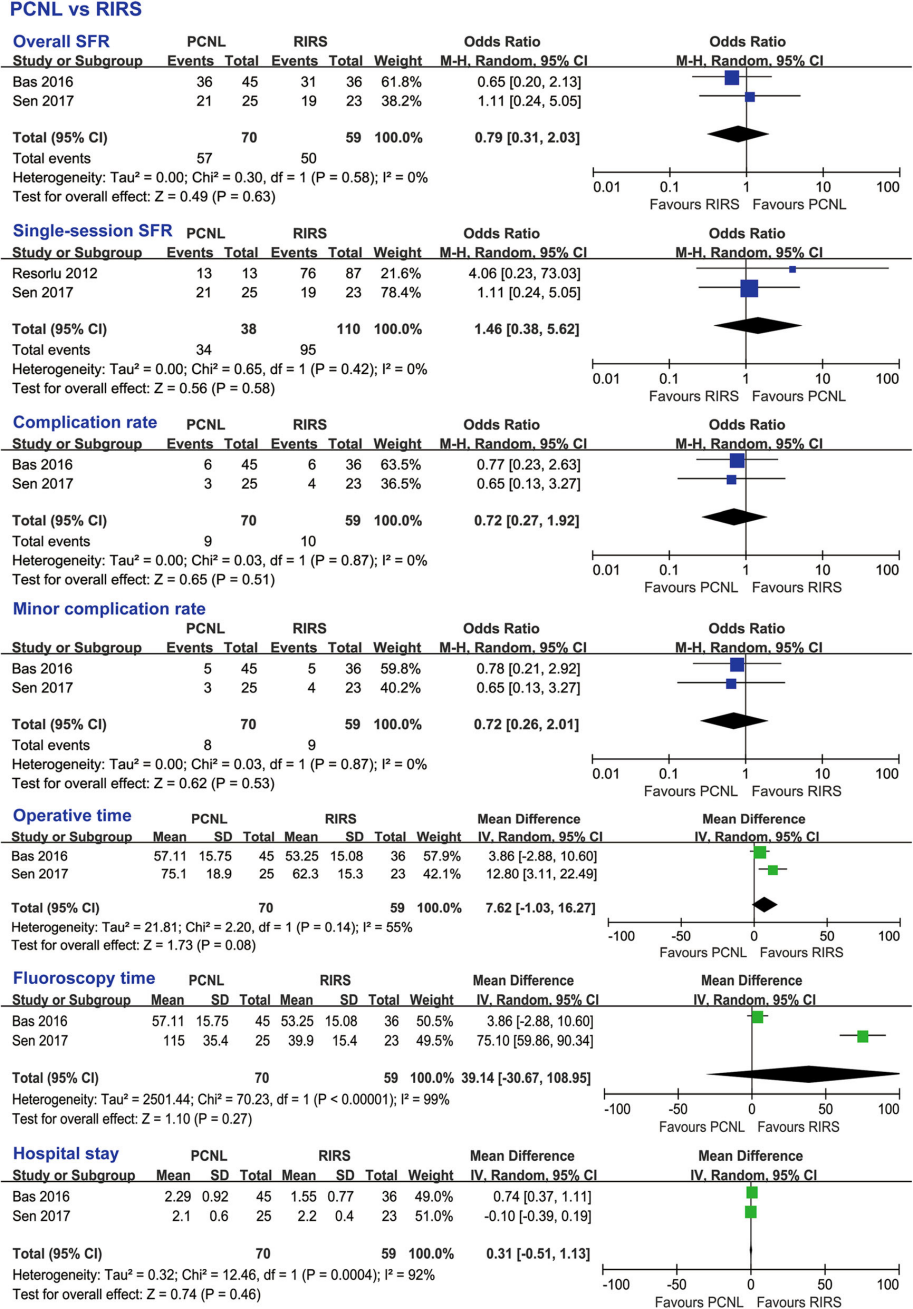
Subgroup analyses results of PCNL vs RIRS

Overall SFR, single-session SFR, complication rate, minor complication rate, operative time, fluoroscopy time and hospital stay were all not significantly different between PCNL and RIRS (Fig. 9).

## Discussion

Pediatric urolithiasis, which has a high risk of a relapse, can currently be managed by SWL, PCNL, RIRS, and laparoscopic or open surgery. Regardless of the selection of treatment modalities, the final objective is to render the pediatric patient stone free and to reduce the recurrence risk to a minimum. The results from this systematic review illustrated that PCNL presented a significantly higher overall SFR (OR 2.69, 95% CI 1.48 to 4.91, *p* = 0.001), higher single-session SFR (OR 4.67, 95% CI 1.68 to 12.98, *p* = 0.003), and higher EQ. (OR 5.49, 95% CI 3.73 to 8.06, *p* < 0.00001) compared with SWL. The RCT of SWL vs PCNL conducted by Kumar A, et al. [17] proved that PCNL was more efficient for stone sizes of 10–20 mm than SWL (EQ: 86.96% vs 53.33%), and the identical conclusion was also reached by the retrospective case controlled studies. Although the pooled results demonstrated the overall SFR and single-session SFR of PCNL were both significantly higher than SWL, there was no significant difference in several studies in terms of the overall SFR (PCNL vs SWL: 96% vs 86.3%; 88% vs 88%; 89.2% vs 88%; 94.9% vs 84.6%) [15, 16, 18, 20]. Considering the lower retreatment rate and auxiliary procedure rate, these studies claimed that PCNL is a feasible and more efficient treatment for pediatric renal stones.

The pooled results revealed there was no significant difference in the SFR between PCNL and RIRS regardless of the stone size of the pediatric patients. Although Resorlu B, et al. [23] also reported that no significant difference of the SFR was found between PCNL and RIRS (94.3% vs 92.6%), if dividing patients into subgroups according to stone size, PCNL was apparently superior to RIRS (> 20 mm: 83.9% vs 50%; < 20 mm: 100% vs 87.3%). Nevertheless, the length of hospital stay was significantly shorter in RIRS pediatric patients in comparison with PCNL (PCNL vs RIRS: 3.1 ± 1.2 vs 1.7 ± 0.6, days) [23]. PCNL requires the creation and dilation of an access tract through the renal parenchyma, and this is deemed to be more invasive than RIRS or SWL. However, the application of micro-PCNL or even super-mini PCNL can reduce complication events with a very high single session SFR.

SWL is considered the most minimally invasive procedure among these 3 treatment modalities, and it is still the first-line therapy for pediatric renal stones. In our analysis, RIRS is a more effective procedure than SWL according to the higher single-session SFR (OR 2.35, 95% CI 1.21 to 4.55, *p* = 0.05) and higher overall SFR in pediatric patients with upper urinary tract stone size ≤20 mm, although comparative studies of SWL vs RIRS included in this review had no positive result in SFR, including from an RCT [28]. Furthermore, the biological effects of SWL may induce acute injury of the renal parenchyma and adjacent tissues due to the acute effects of SWL, such as focal hemorrhage, rupture of small veins, extravasation and pooling of blood, necrosis in vasculature, disintegration in podocytes and mesangial cells, blood within Bowman’s space and renal tubules, ischaemic changes, and infiltration by inflammatory cells [29]. Shock wave-induced transient tubular functional damage has been observed by Villanyi KK and colleagues [30]. Therefore, these authors recommended that consecutive treatments for pediatric renal stones should be spaced by at least 2 weeks. Although there is no clinical evidence about the long-term effect of SWL on pediatric kidneys [29], this effect should be kept in mind when SWL is chosen for pediatric renal stones due to the kidney still being in the growth and development stage.

In this review, higher overall complication rates and higher minor complication rates were only significantly found in the subgroup analyses of PCNL vs SWL. Although there was no significant difference in the various complication rates among the other subgroup analyses, blood transfusion was indicated exclusively in PCNL patients exclusively in our review. The highest transfusion rate, 13.64%, was reported by Saad KS, et al. [24]. Desoky EA and his colleagues [31] claimed that PCNL in the pediatric age group via the flank-free modified supine position was safe and effective in the management of renal pelvis stones of sizes 20–30 cm, and the SFR was similar to conventional PCNL in the prone position with only 1 (4.5%) blood transfusion.

Except for the results of subgroup analyses, there were no significant differences in all of the indexes of the complication rates comparing SWL with the other two treatments. This finding disagrees with the widespread recognition that SWL is the most minimally invasive procedure among these techniques. Moreover, steinstrasse formation was only observed in SWL (1.1–25%), except for 1 case of steinstrasse formation in PCNL [18] and another case in RIRS [22]. However, the fragment clearance after pediatric SWL is more efficient than in adults. D’Addessi A, et al. [28] indicated that, since the child’s ureter is shorter than an adult’s, this shorter length partially compensates for its narrower lumen, and the pediatric ureter is more elastic and distensible, promoting the passage of stone fragments. Finally, the small body volume of children reduces the loss of energy of the shockwaves in the process of release.

SWL had an undoubtedly shorter operative time than PCNL and RIRS, and it is usually performed as an outpatient treatment. All of the included studies preferred SWL over PCNL or RIRS, considering the operative time and hospital stay. However, with instrument miniaturization and other optimizations, RIRS has been increasingly performed, like SWL, as an outpatient procedure. The hospital stay of RIRS was also significantly shorter than PCNL, and this could reduce the medical cost, in spite of no significant difference being found in subgroup analyses. In addition to the relatively longer operative time and longer hospital stay, PCNL also presented a significantly longer fluoroscopy time than SWL or RIRS. Ristau BT and his colleagues [32] reported that radiation exposure was vital during treatment of pediatric stone disease, especially for defining the location of the stone when PCNL was performed. Therefore, these authors recommended that urologists should closely monitor the amount of radiation dose and limit the maximum effective dose to as low as reasonably achievable (ALARA) [33]. Specifically, ALARA principles mandated that the maximum effective dose should not exceed 50 mSv in any 1-year period, and an average dose less than 20 mSv per year over any 5-year period. In brief, the urologists should devote themselves to reducing the radiation hazard as much as possible.

Since SWL is mainly performed under ultrasonographic guidance, it becomes a more attractive option with lower radiation exposure than the other two treatments. However, SWL exhibited a significantly higher retreatment rate compared with the other two therapies, while the retreatment rate between PCNL and RIRS did not show any significant difference. Furthermore, SWL also caused a significantly higher auxiliary procedure rate than PCNL. Considering that the fragment clearance of children is more rapidly than that of adults after SWL, surgical approaches seem to be a last resort. Soygur T and his colleagues [34] suggested that expectant management was usually efficient even in patients who developed steinstrasse after SWL, except for patients with larger stones (> 20 mm), staghorn calculi or sepsis with an obstructed kidney. However, RIRS has a lower efficiency than SWL according to the EQ. In addition, every single EQ of RIRS from the included studies was less than 50% because the complete RIRS process includes one session for passive dilatation, one session for the lithotripsy and stent insertion, and one session for stent removal. Therefore, this technique requires more sessions under anaesthesia and more surgical risks.

When interpreting the results of our review, some limitations should be addressed. First, only 3 RCTs were available for final analysis, and more than half of the included studies were nonrandomized case control comparisons. Inevitably, patients and investigators cannot be double-blinded to the interventions. Allocation concealment was also not described in some RCTs. Furthermore, not all of the included articles were high-quality studies. A lack of more high-quality studies reduces the persuasiveness of our work. Second, no included study compared all three treatments head-to-head, and it was impossible to conduct a high-quality network meta-analysis since there were not enough relevant RCTs. Third, due to the nature of non-randomized studies, selection bias could be a major confounder, which might bias our conclusions. Finally, the heterogeneity among the studies was obvious for several parameters, which might be a result of the differences in pediatric inclusive criteria, physical environment, surgical skills, outcome definitions and standards, or imaging during follow-up. Even more concerning is the stone burdens were not similar among these included studies, and there was not enough data to execute a sufficient subgroup analysis.

In spite of these limitations, our study still has some merits and values. To the best of our knowledge, this is the first systematic review that has simultaneously analysed the efficacy and safety of SWL, PCNL, and RIRS simultaneously in the treatment of pediatric upper urinary tract stones. Furthermore, our work, updated with the most recent data, provides a newly reference for the selection of the ideal treatment modality in pediatric urolithiasis. However, it is noteworthy that making an optimal recommendation is unusually difficult, since the clinical option is based on the stone size, stone location, patient age, instrument caliber, previous interventions history, comorbidity, and other factors.

## Conclusion

In summary, our review suggests that SWL performed as an outpatient procedure provides a shorter hospital stay and operative time, with a lower SFR, higher retreatment rate, higher auxiliary procedure rate, and relative lower EQ. PCNL presents a higher SFR than SWL and the highest EQ, but this is a technique accompanied by a longer fluoroscopy time and operative time than the other two modalities. RIRS offers a higher single-session SFR and lower retreatment rate than SWL but a lower EQ than SWL and PCNL, although a significantly shorter hospital stay than PCNL. Higher complication rates and higher minor complication rates were only significantly found in subgroup analyses of PCNL vs SWL. In other words, the complication rates are comparable among the three modalities, and most of these were minor complications.

The ultimate target of pediatric urinary stone management is to achieve extreme stone clearance with a safe and effective therapeutic regimen. To achieve this ideal target, urologists must choose the optimal individual modality, or combined other therapeutic regimens, according to the pediatric conditions and the goals of the parents.

## Data Availability

All data analysed during this study are included in this published article.

## Abbreviations

ALARA: As low as reasonably achievable
AUA: American Urological Association
CI: Confidence interval
EAU: Uropean Association of Urology
EQ: Effectiveness quotients
GFR: Glomerular filtration rate
LE: Level of evidence
NOS: Newcastle-Ottawa Scale
OR: Odds ratio
PCNL: Percutaneous nephrolithotomy
RCT: Randomized control trail
RIRS: Retrograde intrarenal surgery
SD: Standard deviation
SFR: Stone free rate
SWL: Extracorporeal shockwave lithotripsy
WMD: Weighted mean difference
